# Peri-implant bone preservation of a novel, self-cutting, and fully tapered implant in the healed crestal ridge of minipigs: submerged vs. transgingival healing

**DOI:** 10.1007/s00784-021-03970-0

**Published:** 2021-05-05

**Authors:** Helena Francisco, Gary Finelle, Fabien Bornert, Rebecca Sandgren, Valentin Herber, Nils Warfving, Benjamin E. Pippenger

**Affiliations:** 1 Faculdade de Medicina Dentária, Universidade da Lisboa (University of Lisbon), Lisbon, Portugal; 2 Private practice, Paris, France; 3 Department of Oral Surgery, University of Strasbourg, Strasbourg, France; 4 Biomedical Center, Lund University, Lund, Sweden; 5 Department of Dentistry and Oral Health, Division of Oral Surgery and Orthodontics, Medical University of Graz, Graz, Austria; 6 AnaPath Services AG, Liestal, Switzerland; 7 Department of Preclinical & Translational Research, Institut Straumann AG, Basel, Switzerland; 8 Department of Periodontology, Center for Dental Medicine, University of Bern, Bern, Switzerland

**Keywords:** Hard bone, Healed crestal ridge, Roxolid®, Tapered implant, Crestal bone preservation, Transgingival, Submerged

## Abstract

**Objectives:**

The aim of this study was to assess the influence of transgingival compared with submerged healing on peri-implant bone maintenance around a novel, fully tapered implant in a healed crestal ridge in minipigs.

**Materials and methods:**

In each of 12 minipigs, two implants (Straumann® BLX, Roxolid® SLActive®, Ø 3.75 × 8 mm) were placed. Implants were either left for submerged or for transgingival healing for 12 weeks. Measurements performed were bone-to-implant contact (BIC), first bone-to-implant contact (fBIC), bone area to total area (BATA), perpendicular bone crest to implant shoulder (pCIS), bone height change from placement, and bone overgrowth (for submerged implants).

**Results:**

No significant differences were found between transgingival and submerged healing in any of the measured parameters, except for BATA on the buccal aspect in which significantly more bone formation was found for the transgingival healing group. For both groups, there was a gain in crestal bone height during the 12-week healing period.

**Conclusions:**

Loaded compared with unloaded implants displayed comparable levels of osseointegration and equivalent marginal bone levels. This qualifies the implant placement protocol with respect to the osteotomy dimensions and subcrestal placement protocol for immediate loading.

**Clinical relevance:**

The here presented results related to osseointegration and crestal bone maintenance after submerged or transgingival healing have demonstrated a high level of consistency in the used in vivo translational model. The obtained results support the translation of the novel implant type in conjunction with the developed surgical workflow and placement protocol into further clinical investigation and use.

## Introduction

In recent years, implant dentistry has seen a distinct optimization of the implant treatment procedure. Recent trends focus on early and immediate procedures, which today outperform the number of more conservative late procedures [1]. Moreover, treatments are increasingly extended into compromised patient populations displaying limited bone quality or quantity [1–3]. This trend has been driven by increasing patient demands in aesthetic and immediate function and by the increasing willingness of clinicians to use implants in a wide variety of challenging clinical situations [4, 5].

Primary stability is a vital factor when considering the use of implants in challenging situations, particularly when shorter treatment times, and therefore accelerated healing times, are called for. Long-term success in these circumstances relies on good primary stability at implant placement, since it plays a key role in achieving good osseointegration [6]. Inadequate or sub-optimal stability can lead to micro-motion of the implant, which compromises the osseointegration process and can lead to implant failure [7, 8]. Maximum insertion torque is important in achieving primary implant stability, particularly for immediate implant loading procedures [9, 10]. Insertion torque is influenced by the drill protocol, e.g., the diameter and type of drill employed for implant placement [11, 12], and also by the geometry and design of the implant, e.g., the implant shape and the type of thread configuration [13].

Advances in dental implant surface technology over the last 20 years have also given clinicians a major boost in the ability to treat challenging situations. Modern implant surface treatments, including changes in surface topography and chemistry, have been designed to make osseointegration faster and stronger and also maintain the crestal bone [14, 15]. Other strategies to optimize dental implant treatment have included shortening the implant healing time, e.g., by loading the implant immediately (avoiding a two-stage intervention) or early after placement. Clinical evidence has shown no difference in success and survival rates between immediately loaded and conventionally loaded implants [16, 17], and there is some evidence for bone preservation with immediate loading [18, 19].

Recently a new self-cutting tapered implant design to maximize primary stability in situations with limited bone quantity has been proposed [20]. This novel implant design displays a specifically protruding thread geometry combined with a reduced diameter implant neck, which significantly defines its mechanism of bone engagement and force distribution under loading conditions. The aim of this controlled preclinical study was therefore to compare the effect of disparate loading regimes resulting from either submerged or transgingival healing on marginal bone levels, implant osseointegration, and peri-implant bone formation around this novel implant type in the context of the developed osteotomy preparation technique and placement characteristics in a standardized fully healed porcine mandibular model. The primary objective of this non-randomized controlled preclinical pilot-study was to compare crestal bone level changes around aBLXBLX novel fully tapered, self-cutting bone level implant under two healing regimes, i.e., submerged (control) and transgingival (test) healing as part of a non-inferiority study design. Secondary objectives addressed the comparison of osseointegration and peri-implant bone formation.

## Materials and methods

### Study design

This study was performed at the Biomedical Department of Lund University, Sweden, after ethical approval (ethical permit number M-192-14) and performed in accordance with the Swedish Animal Protection Law and ISO 10993-6 (Biological evaluation of medical devices – Part 6: Tests for local effects after implantation). Reporting followed the ARRIVE (Animal Research: Reporting of In Vivo Experiments) guidelines regarding all relevant items [21].

Twelve adult female Göttingen Minipigs™ (Ellegaard Göttingen Minipigs A/S, Dalmose, Denmark) of age between 20 and 24 months at the time of surgery and an average body weight of ca. 40 kg were included in this study. Comparisons were performed in a standardized, fully healed edentulous mandibular minipig model using a single-end point after 12 weeks of healing. A total of 24 implants (Straumann BLX® - SLActive® surface, 3.75 mm diameter × 8 mm long, Institut Straumann AG, Basel Switzerland), i.e., 12 implants per group and 1 test and control implant per animal were tested in a lateral arrangement in Goettingen^TM^ Minipigs. In order to mitigate potential effects of implant position on histological outcomes, implantation sites of individual implant types were rotated and to side switched from animal to animal. Primary parameters related to marginal bone level changes were the distance from the implant shoulder to the first bone to implant contact (fBIC) and the perpendicular bone crest to implant shoulder (pCIS). Secondary parameters included the coronal and apical bone to implant contact (BIC), the bone area to total area (BATA), and bone overgrowth (BOG) for submerged implants, respectively.

The animals were kept in pens, in groups of three with a minimum of 1 week to acclimatize to the environmental conditions prior to surgery. They were fed a standard soft food diet (Special Diet Services (SDS), Witham, UK #801586) and fasted overnight prior to surgery to prevent vomiting.

### Surgical procedure

Surgical interventions were performed under aseptic conditions in an operating suite dedicated to animal surgery. General anesthesia was used for all surgical procedures by means of an intramuscular injection. A combination of dexmedetomidine (25 – 35 μg/kg, Dexdomitor®, Orion Pharma Animal Health, Espoo, Finland) and tiletamine-zolazepam (50 – 70 mg/kg, Zoletil® 100 vet, Virbac, Carros, France) were used and maintained by intravenous infusion until effect with propofol (PropoVet® Multidose, Orion Pharma Animal Health, Espoo, Finland) and fentanyl (B.Braun Fentanyl, Melsungen, Hessen, Germany).

Prophylactic Carprofen (4 mg/kg s.i.d., i.m. Rimadyl® Vet., Orion Pharma Animal Health, Espoo, Finland) was provided. Post-surgically, for up to 4 days, Carprofen (4 mg/kg s.i.d., i.m. Rimadyl Vet., Orion Pharma Animal Health, Espoo, Finland) was given together with buprenorphine (0.03 mg/kg, i.m. Vetergesic® Vet, Orion Pharma Animal Health, Espoo, Finland). Intra-operatively, an infiltrative injection with 1.8 ml of Xylocaine (Xylocain Dental adrenalin 20 mg/mL + 12.5 mg/mL; Astra AB, Södertälje, Sweden) per hemi-mandible was used to provide local anesthesia. The animals were intubated during anesthesia, and breathing was controlled by a ventilator. Vital parameters were monitored continuously during surgery.

Animals were treated prophylactically with antibiotics—benzylpenicillin procaine-dihydrostreptomycin (25 mg/kg plus 20 mg/kg, s.i.d., i.m. (Streptocillin vet, Boehringer Ingelheim International GmbH, Ingelheim am Rhein, Germany)—and for 7 days post-operatively (Streptocillin vet, 3-4 mL/pig i.m., Boehringer Ingelheim International GmbH, Ingelheim am Rhein, Germany). Further analgesia was given if necessary when monitoring the animals during the healing phase.

### Tooth extraction

Preparation of test sites was based on careful extraction of contralateral mandibular premolars (P2-P4) and first mandibular molars (M1) under general anesthesia via a minimally invasive surgical approach, i.e., without raising a flap.

### Osteotomy creation and implant placement

**Fig. 1:**
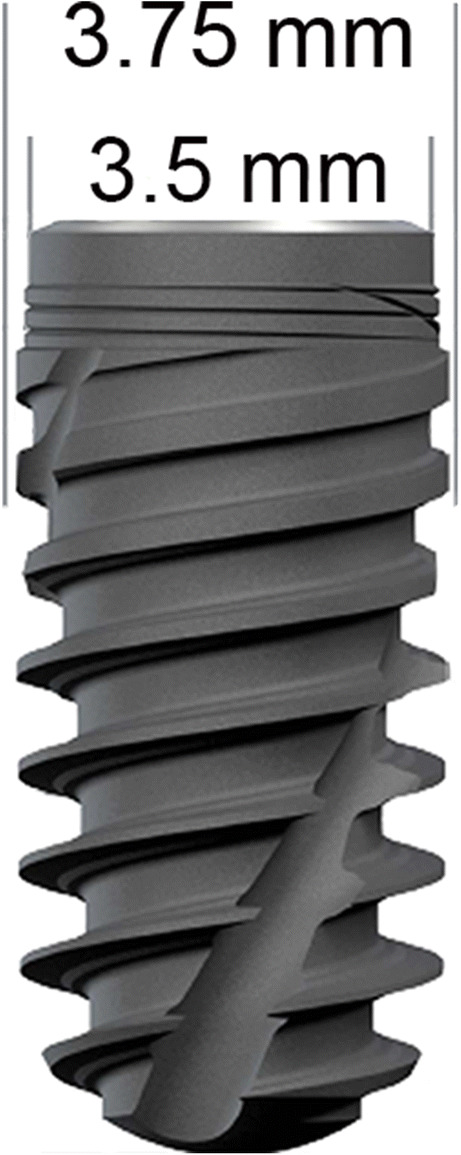
Image of implant body design including measurements; the self-cutting nature of the implant can clearly be seen (figure used with permission of the Straumann Group)

After 3 months of healing, the mandibular alveolar ridge was exposed on both sides after crestal incision and reflection of the muco-periosteal flap. Gentle bone grinding was performed under constant cold saline cooling to create an even ridge with a width of at least 8 mm. Novel fully tapered implants (BLX, Roxolid® SLActive® Ø 3.75 mm × 8 mm long) (Fig. 1) were placed using novel drills, specifically designed for this type of implant, and a simplified drill protocol that includes less drill steps and uniform drill speeds compared with the conventional Straumann® protocol. A drill protocol for hard bone was employed, consisting of an initial needle drill followed by 2.2, 2.8, 3.5, and 3.7 mm (only crestal 4 mm) diameter drills. No tapping was applied. The implant axis was checked with an alignment pin in between each drill diameter increase. Implants were placed by hand using a ratchet with a torque scale of 0–80Ncm (Straumann Group, Basel, Switzerland). The remaining available hemi-mandibular bone slots were also implanted for the purposes of other studies not within the scope of the present study (a total of 8 implants per mandible were placed—2 implants per mandible are presented herein). A pre-allocation implant placement scheme was applied to ensure that both groups were intentionally placed in the posterior mandibular P4/M1 region to minimize the effect of bone density as a parameter influencing the study.

The implants were placed 1-mm subcrestally. A closure screw (Straumann Group, Basel, Switzerland) or a healing abutment (BLX healing abutment, gingival height 2.5 mm, abutment height 2 mm, Straumann Group, Basel, Switzerland) was then allocated to either implant and flaps were repositioned (either full closure or around the transgingival abutment, respectively) such that primary closure was achieved. Interrupted sutures were used to optimize robust healing (Vicryl® 5.0, Ethicon, USA). Photographs were taken using a Nikon D5300 (Nikon, Tokyo, Japan) from all sites after implant placement and at termination. For each implant, the maximum insertion torque (max IT) was measured and recorded at the time of implant placement.

**Fig. 2:**
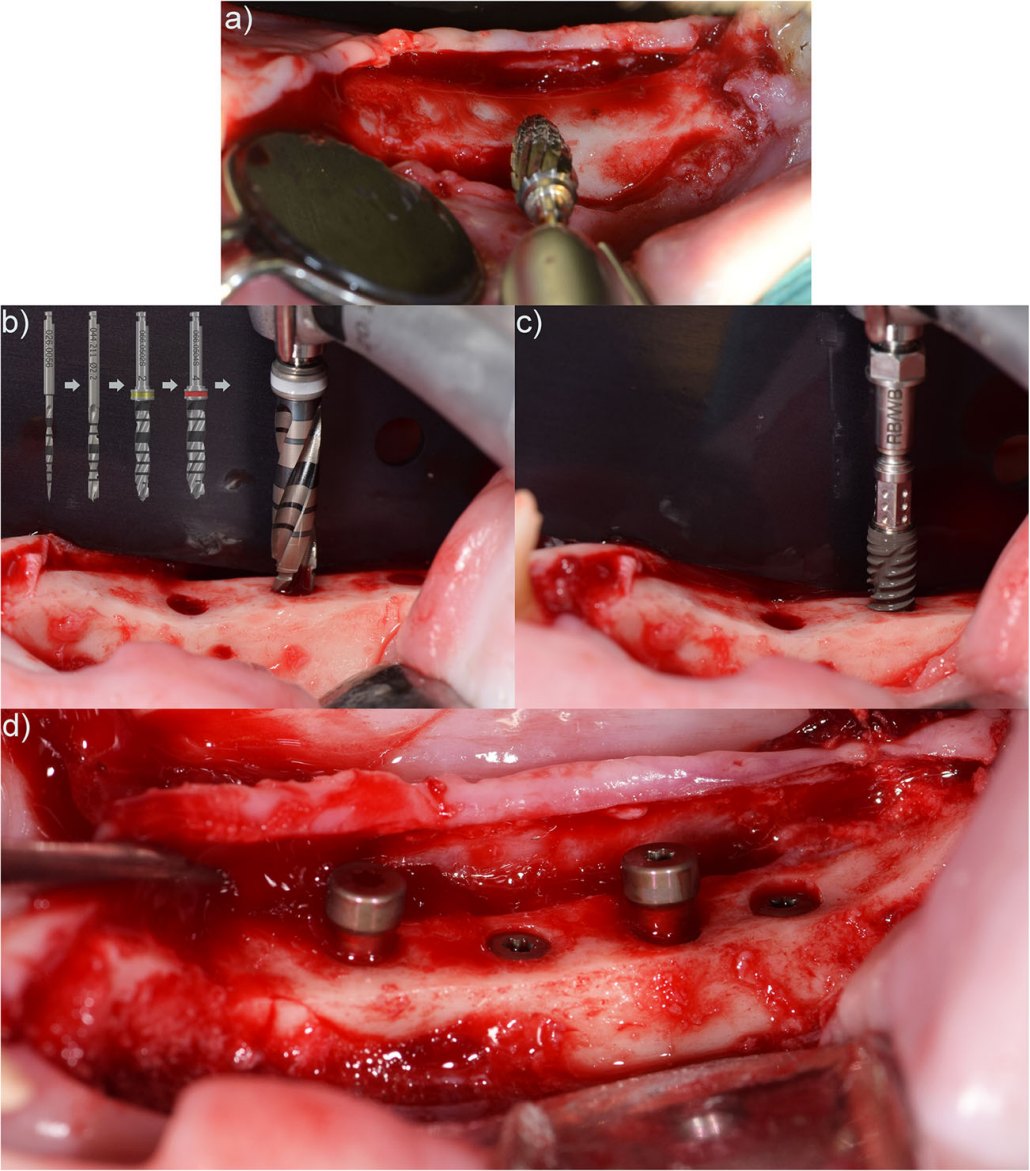
Images taken during implant placement representing the overall surgical workflow. (**a**) Ridge flattening, (**b**) implant bed preparation (additional drill steps presented in line with captured drill step), (**c**) implant placement, and (**d**) placed implants with healing caps or healing abutments

The overall surgical procedure is visualized in Fig. 2.

### Termination

All animals were sacrificed 12 weeks after surgery by means of an intravenous injection of pentobarbital (20% solution of Pentobarbital-natrium, Apoteket AB, Stockholm, Sweden, 60 mg/ml) to induce cardiac arrest.

### Histological processing

Block resectioning of the hemi-mandible implant sites was performed using an oscillating autopsy saw, leaving the soft tissue intact. Fixation was performed by immersing the hemi-mandibles in formalin (formaldehyde 4% solution) for a minimum of 2 weeks before histological processing.

**Fig. 3:**
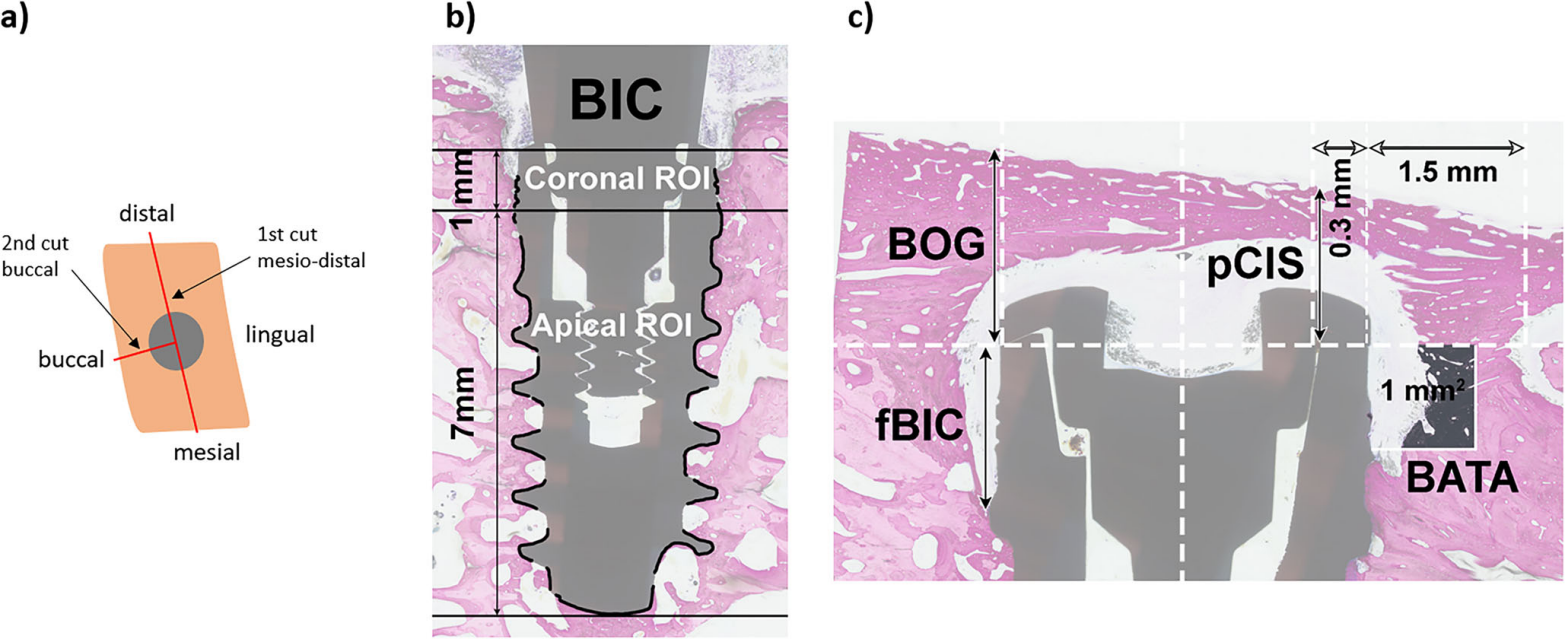
Histological sectioning and explanation of histomorphometric measurements

The bone samples were firstly immersed in formalin buffer solution, then, using ascending grades of alcohol and xylene they were dehydrated, followed by the use of methyl methacrylate for infiltrating, embedding and non-decalcified sectioning. Each target site was cut first in mesio-distal and then in buccal direction resulting in a half-buccal section and a full mesio-distal section, each of roughly 500 μm in thickness (Fig. 3a). The sections were ground to a final thickness of 30–50 μm and stained with paragon stain (toluidine blue and basic fuchsin).

### Histomorphometric analysis

The histomorphometric measurements were performed as follows (Fig. 3b,c):

- fBIC was measured on three sides: mesial, distal, and buccal.
- pCIS was measured on all three sides (mesial, distal, and buccal). Therefore, a ROI was defined: 1.5 mm distally, mesially, and buccally from the implant shoulder. A perpendicular line from the implant center axis to the most coronal border of bone tissue within the ROI was drawn and the distance from this line to the implant shoulder was measured.
- BIC was measured in two regions of interest (ROI)—Coronal 1 mm and Apical 7 mm, measured from the implant shoulder on all three sides (mesial, distal and buccal).
- BATA was calculated within a 1 × 1 mm area at the coronal part of the implant.
- BOG was measured on the mesial, distal, and buccal sides at a line from the implant shoulder parallel to the longitudinal implant axis. The vertical distance from the implant shoulder to the coronal intersection with bone on this line was measured.

### Statistical analysis

Descriptive statistics (mean, standard deviation (SD), median, and interquartile range) were calculated for all measured outcomes, with stratification by treatment (submerged vs. transgingival healing). To examine the difference between treatments, first, the Wilcoxon signed rank test (paired *t* test) for paired comparisons was used.

For non-inferiority testing between test and control groups, multivariate regression analysis with the factor “animal” introduced in the models as a random effect was performed. The Dunnett-Hsu adjustment [22] was used to adjust the *p* values in the case of multiple comparisons. The average effect (difference between the adjusted means of the two implants) and its two-tailed 90% confidence interval (equivalent to a one-tailed 95% CI (CI), to test for non-inferiority) were calculated, as a measure of how different (i.e., the tolerance range) the outcomes between the two protocols were. The lower 95% CI yielded the tolerance range necessary to state non-inferiority [23]. An a priori sample size calculation was not performed, but the sample size of 12 was chosen as appropriate to assess certain biological reactions in a preclinical study. A post hoc sample size analysis demonstrated that the sample size of 12 animals resulted in a study power of 63%. This assumes that the mean BIC coronal for the submerged would be 62.80 and 69.60% for the transgingival implant group with a standard deviation of 18.90 and that a difference of 9% or less is unimportant. Alpha (1 tailed) was set to 0.05. All statistical analyses were performed using SAS software (version 9.4. 2016, SAS Institute Inc., Cary, NC, USA). Linear mixed models were fit using the algorithm PROC MIXED which uses the method of restricted maximum likelihood (also known as residual maximum likelihood).

## Results

All animals recovered well from the surgical procedures and displayed uneventful healing. No signs of surgical, peri-operative or post-operative complications, peri-implant inflammation or peri-implant bone dehiscence or loss were observed.

### Insertion torque

IT values between test ((28.88 ± 8.31) Ncm) and control ((29.83 ± 9.04) Ncm) groups were comparable (*p* = 0.6629).

### Descriptive histology

**Fig. 4:**
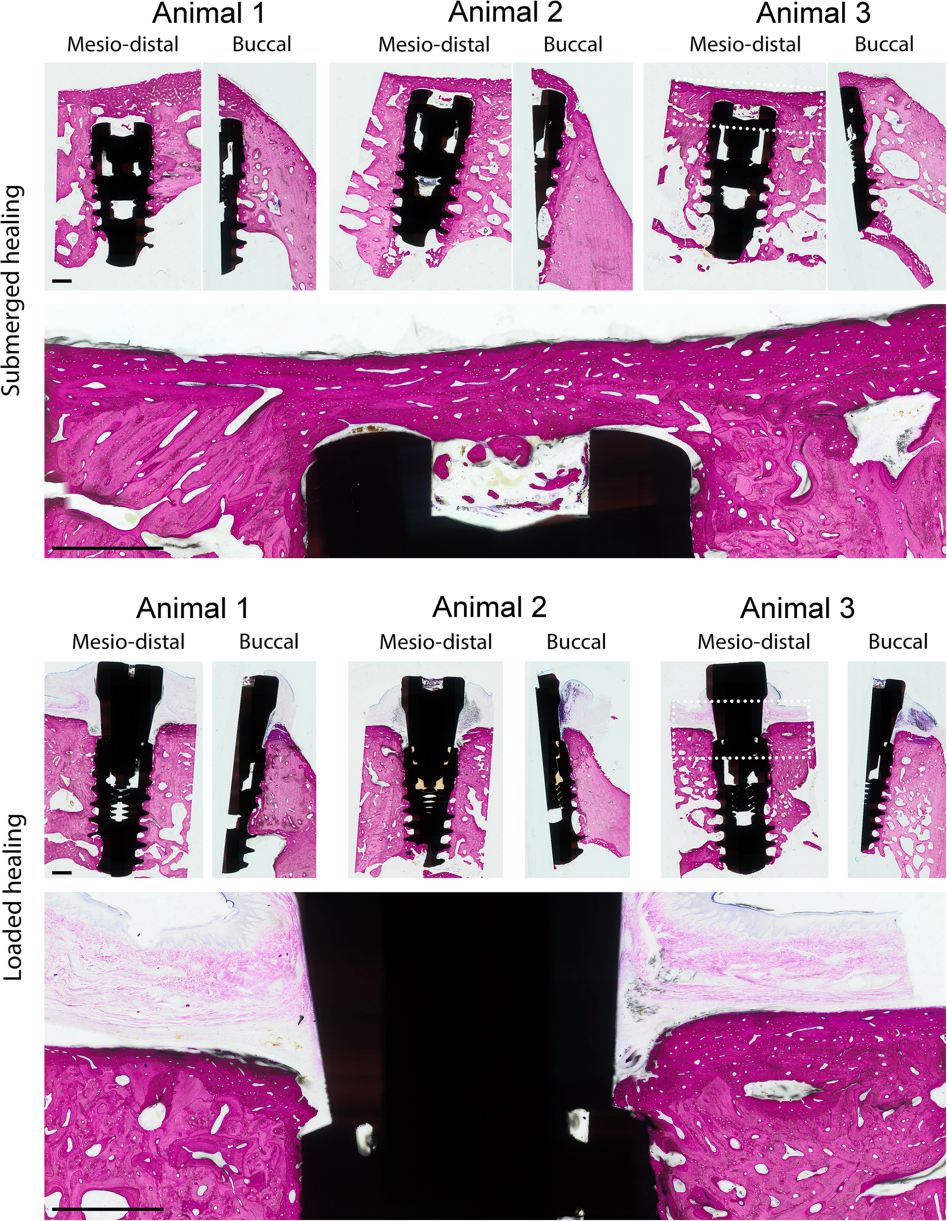
Representative histological images after 12 weeks of healing. Images in which the full implant is captured are mesio-distal slices; images in which half of the implant is captured are buccal half slices. Dotted white boxes contain area for presented zoomed images presented directly below each boxed image. Each row has its own scale bar. All scale bars = 1 mm

As evidenced by the histological micrographs in Fig. 4, all test and control implants were well osseointegration after 12 weeks. The healing pattern of submerged implants was consistently characterized by a dense layer of cortical bone overgrowing the coronal aspect of the implant and healing cap in close contact along the complete perimeter of the implant and healing cap. This pattern was consistently observed for all submerged implants (*n* = 12). The healing profile of transgingivally healed implants was characterized by the appearance of a step-like marginal bone profile displaying an almost vertical gap parallel to the abutment interface that was consistently apically confined to the coronal shoulder of the implant platform. New vertical bone growth onto the bevel of the implants could be observed (Fig. 4 lower image). The apical portion of the implants in both groups was consistently characterized by bony trabeculae forming mainly at the thread tip of the implants that extended into the cancellous marrow.

### Histomorphometric analyses

Tables 1 and 2 report the descriptive statistics of the histomorphometric parameters as a function of healing modality as well as the statistical associations between these parameters and study groups as derived from mixed regression analysis respectively. BLX Table 2 reports the results from the non-inferiority comparisons of the study groups.

**Table 1:** Descriptive statistics and paired unadjusted comparisons (Wilcoxon signed rank test) for the outcomes stratified by study groups

Outcome	Transgingival healing (*n* = 12) Mean ± SD Median (IQR)	Submerged healing (*n* = 12) Mean ± SD Median (IQR)	Mean _diff_ ± SD _diff_	Wilcoxon signed rank test *p* value
BIC Apical ROI (7 mm) [%], mesio-distal	62.5 ± 10.3	66.1 ± 12.9	−3.6 ± 13.9	0.4697
	64.4 (53.4 to 67.5)	72.2 (56.0 to 74.4)		
BIC Apical ROI (7 mm) [%], buccal	61.6 ± 15.5	58.1 ± 18.1	3.5 ± 16.3	0.5693
	62.4 (52.0to 75.7)	60.2 (47. to 71.)		
BIC Coronal ROI (1 mm) [%], mesio-distal	54.2 ± 14.0	62.9 ± 19.9	−8.7 ± 26.0	0.1294
	53.0 (47.0 to 62.3)	63.6 (51.3 to 82.6)		
BIC Coronal ROI (1 mm) [%], buccal	69.6 ± 20.9	62.8 ± 18.9	6.8 ± 31.8	0.5693
	74.5 (52.2 to 86.1)	68.7 (55.3 to 73.3)		
fBIC [μm], buccal	−99.8 ± 219.7	0 ± 0	−99.8 ± 219.7	0.1250
	0 (−75.07 to 0)	0 (0 to 0)		
fBIC [μm], mesial	−61.7 ± 98.9	−5.8 ± 19.9	−55.9 ± 104.6	0.0938
	0 (−105.2 to 0)	0 (0 to 0)		
fBIC [μm], distal	−55.0 ± 98.0	0 ± 0	−54.9 ± 98.0	0.1250
	0 (−75.9 to 0)	0 (0 to 0)		
BATA [%], buccal	86.4 ± 4.7	81.8 ± 7.3	4.6 ± 6.9	0.0342
	87.0 (83.2 to 89.6)	81.8 (77.1 to 86.5)		
BATA [%], mesial	82.4 ± 5.9	80.4 ± 8.5*	1.1 ± 8.7	0.6377
	82.6 (77.9 to 85.9)	82.8 (71.7 to 89.5)		
BATA [%], distal	82.3 ± 4.9	84.5 ± 5.3*	−2.5 ± 4.0	0.1016
	83.7 (79.5 to 85.1)	85.5 (81.1 to 87.9)		
pCIS [μm], buccal	1439.7 ± 608.4	1700.0± 602. 1	−260.3 ± 610.4	0.1099
	1447.3 (997.7 to 1797.2)	1708.4 (1250.3 to 2103.1)		
pCIS [μm], mesial	1401.8 ± 419.0	1486.5 ± 591.0	−84.7 ± 712.3	0.9697
	1197.0 (1111.0 to 1766.3)	1430.3 (1075.8 to 1898.9)		
pCIS [μm], distal	1552.8 ± 634.1	1911.41 ± 872.803	−358.6 ± 1094.9	0.2036
	1469.7 (1210.4 to 1736.2)	2065.4 (1325.3 to 2398.3)		

*n = 11 for submerged healing for BATA [%] mesial and BATA [%] distal

Difference comparison = transgingival healing—submerged healing

*SD* standard deviation, *IQR* interquartile range (from first to third quartile), *ROI* region of interest, *BIC* bone to implant contact, *fBIC* first bone to implant contact, *BATA* bone area to total area, *pCIS* perpendicular bone crest to implant shoulder

**Table 2:** Association between each outcome and treatment type (*n* =12 analysis unites) corrected for the effect of the animal. The average effect and non-inferiority test are also given

Outcome	Factor	Value	Regression parameters			Adjusted^§^ parameters for multiple comparisons			Non-Inferiority	
			Estimate	SE	(*t* test)_Reg_ *p* value	Adjusted^§^ mean	95% CI for the adjusted mean	Dunnett-Hsu *p* value	Average effect of the factor	Lower 90% CI^†^
BIC Apical ROI (7 mm) [%], mesio-distal	Intercept		66.1	3.4	< .0001					
	Treatment	Transgingival	−3.6	4.0	0.3869	62.5	55.1–69.9	0.3869	−3.6	−10.9 to 3.6
		Submerged	0.0			66.1	58.7–73.6	Ref.		
BIC Apical ROI (7 mm) [%], buccal	Intercept		58.1	4.9	< .0001					
	Treatment	Transgingival	3.5	4.7	0.4725	61.6	50.8–72.3	0.4725	3.5	−4.9 to 11.9
		Submerged	0.000			58.1	47.3–68.8	Ref.		
BIC Coronal ROI (1 mm) [%], mesio-distal	Intercept		62.9	5.0	< .0001					
	Treatment	Transgingival	−8.7	7.0	0.2429	54.2	43.3–65.2	0.2429	−8.7	−21.3 to 4.0
		Submerged	0.0			62.9	52.0–73.9	Ref.		
BIC Coronal ROI (1 mm) [%], buccal	Intercept		62.8	5.8	< .0001					
	Treatment	Transgingival	6.8	8.1	0.4191	69.6	57.0–82.7	0.4191	6.8	−7.8 to 21.4
		Submerged	0.0			62.6	50.1–75.5	Ref.		
fBIC [μm], buccal	Intercept		0.0	44.8	0.9999					
	Treatment	Transgingival	−99.8	63.4	0.1436	−99.8	−198.5 to −1.2	0.1436	−99.8	−213.7 to 14.0
		Submerged	0.0			0	−98.7 to 98.7	Ref.		
fBIC [μm], mesial	Intercept		−5.8	20.6	0.7850					
	Treatment	Transgingival	−55.9	29.1	0.0812	−61.6	−107.0 to −16.3	0.0812	−55.9	−108.2 to 3.6
		Submerged	0.0			−5.8	−51.1 to 39.6	Ref.		
fBIC [μm], distal	Intercept		0.000	20.0	1.0000					
	Treatment	Transgingival	−54.9	28.3	0.0782	−54.9	−99.0 to −10.9	0.0782	−54.9	−105.8 to 4.1
		Submerged	0.0			0	−44.0 to 44.0	Ref.		
BATA [%], buccal	Intercept		81.8	1.8	< .0001					
	Treatment	Transgingival	4.6	2.0	0.0422	86.4	82.5–90.3	0.0422	4.6	1.0–8.1
		Submerged	0.0			81.8	77.9–85.7	Ref.		
BATA [%], mesial	Intercept		80.7	2.2	< .0001					
	Treatment	Transgingival	1.7	2.6	0.5284	82.4	77.7–87.0	0.5284	1.7	−3.0 to 6.5
		Submerged	0.0			80.7	75.8–85.5	Ref.		
BATA [%], distal	Intercept		84.7	1.5	< .0001					
	Treatment	Transgingival	−2.4	1. 2	0.0690	82.3	79.0–85.5	0.0690	−2.4	−4.6 to 0.3
		Submerged	0.0			84.7	81.4–88.0	Ref.		
pCIS [μm], buccal	Intercept		1700.0	174.7	< .0001					
	Treatment	Transgingival	−260. 3	176.2	0.1677	1439.7	1055.1–1824.2	0.1677	−260.3	−576.7 to 56.2
		Submerged	0.0			1700.0	1315.4–2084.5	Ref.		
pCIS [μm], mesial	Intercept		1486.5	147.9	< .0001					
	Treatment	Transgingival	−84.7	205.6	0.6883	1401.8	1076.4–1727.3	0.6883	−84.7	−454.0 to 284.6
		Submerged	0.0			1486.6	1161.1–1812.0	Ref.		
pCIS [μm], distal	Intercept		1911.4	220.2	< .0001					
	Treatment	Transgingival	−358.6	311.4	0.2740	1552.8	1068.2–2037.5	0.2740	−358.6	−918.0 to 200.7
		Submerged	0.0			1911.4	1426.7–2396.1	Ref.		

§The factor animal was introduced in the mixed linear regression model as a random effect. This effect is statistically significant only for BA/TA distal (*p* = 0.0301). Number of analysis units = 12; number of observations in regression = 24

†Two tailed

*Ref.* Reference level for the comparison within a factor, *SE* standard error, *ROI* region of interest, *BIC* bone to implant contact, *fBIC* first bone to implant contact, *BATA* bone area to total area, *pCIS* perpendicular bone crest to implant shoulder

Apical and coronal BICs around submerged and transgingivally healed implants were consistently comparable, non-inferior, and did not display any significant differences on either of the mesio-distal or buccal aspects of the implant. Specifically, apical values ranged between 58.1% (CI: 47.3% to 68.8%) and 66.1 % (CI: 58.7% to 69.9%), which were registered for the buccal and mesio-distal aspects of submerged implants, respectively. Coronal BIC values were similar and displayed values between 54.2% (CI: 43.3% to 65.2%) and 69.6% (CI: 57.0% to 82.7%) as measure for the transgingival group on the mesio-distal and buccal aspects respectively.

Coronal bone levels as evaluated in terms of fBIC were consistently lower but non-inferior on all, i.e., buccal, mesial, and distal aspects after transgingival healing when compared with submerged healing. Differences reached only borderline statistical significance for the mesial and distal aspects, respectively. Specifically differences between the transgingival and submerged groups were most pronounced on the buccal aspect (−99.8 μm vs. 0 μm, *p* = 0.1436), followed by the mesial (−61.6 μm vs. −5.8 μm, *p* = 0.0812) and distal aspects (−54.9 vs. 0 μm, *p* = 0.0782).

Interestingly BATA values on the buccal aspects were statistically significantly higher around transgingivally healed implants when compared with implants after submerged healing (transgingival: 86.4% (CI: 82.5% to 90.3%) vs. submerged: (81.8% (CI: 77.9% to 85.7%), *p* = 0.0422). BATA values were however comparable on the mesial (*p* = 0.5284) and on the distal (*p* = 0.0690) aspects and ranged between 80.7 (CI 75.8% to 85.5%) and 84.7% (CI 81.4% to 88.0%), which were both measured for the submerged implants and on the mesial and distal aspects, respectively. Neither of the BATA-related parameters were non-inferior when comparing both healing modalities.

Crestal bone levels as evaluated in terms of pCIS were consistently higher but again non-inferior around submerged implants when compared with implants after transgingival healing. None of the evaluated differences in pCIS between healing modalities reached statistical significance. Specifically values ranged between 1401.8 μm (CI: 1076.4 μm to 1727.3 μm) as measured on the mesial aspect of the transgingival group and 1911.4 (CI: 1426.7 μm to 2396.1 μm) for the distal aspect of the submerged group, which were all higher compared with the theoretical initial subcrestal placement level of 1000 μm and which indicated an overall and comparable bone height gain in both groups.

BOG values of the submerged implants were generally comparable to pCIS values and ranged between 1694.3 ± 522.1 μm on the buccal aspect and 1480 ± 609.5 μm on the mesial aspects of the implants.

## Discussion

This pilot study examined the healing and osseointegration of a novel implant type. Implants were placed 1 mm subcrestally in an implant osteotomy that can be considered as apically underprepared with regard to implant thread diameter (Ø3.75 vs Ø3.5 for the osteotomy) and coronally overprepared with regard to the tapered implant body and implant neck (Ø3.5 mm vs Ø3.7 for the osteotomy) respectively. As a consequence, the question arises as to implant stability upon initial placement and during healing and as to the characteristics of bone healing and bone integration given a presumably different stress distribution at the apical and coronal aspects of the implant under different loading regimes. For this reason, a side by side comparison was used to compare the osseointegration of this novel implant type under different healing (submerged vs. transgingival) modalities, which translated into different loading regimes in regard to histomorphometric marginal bone level changes, BIC, and bone density around the implants. Hereby the term loading might be associated more to a mechanical stimulation of the implant from masticatory and lingual forces by contrast to the application of occlusal forces in a traditional sense [24, 25]. Implant stability was verified for the two groups in terms of insertion torque measurements indicating comparable and consistent primary implant stabilities and indirectly consistent implant osteotomy preparation and implant placement conditions for both study groups.

Implant insertion torque and primary implant stability are influenced by a number of different factors including bone quality and density, implant geometry, osteotomy dimensions, and osteotomy preparation technique, all of which ultimately influence the “press-fit” during implant insertion [26]. The tested implant might display a more unconventional mechanism of bone engagement that is mainly based on the widely protruding self-cutting threads. Specifically, the available volume between these threads decreases in the coronal direction and therefore compacts and densifies the peri-implant bone during insertion. Under consideration of the relative osteotomy and implant dimensions, the observed insertion torque and primary stability might therefore mainly result from bone engagement at the apical and central aspects of the implant and not from a more conventional interaction between its coronal part and dense cortical bone. This might also be reflected by the relatively moderate to low insertion torques when compared with values that have been reported for more traditional implant designs in the same animal model [24].

Related to this specific bone engagement mechanism, it was unknown how this potentially different distribution of immediate mechanical loads along the apico-coronal axis of the implant would influence osseointegration and marginal bone levels. For this reason, intra-implant (coronal vs apical) and inter-implant (transgingival vs. submerged) comparisons of osseointegration and bone densities were performed in addition to the evaluation of marginal bone levels as assessed by fBIC and pCIS. Specifically, BIC values were comparable between both healing modalities for the individual apical and the coronal regions, respectively. This indicates that transgingivally healed, loaded implants displayed sufficient primary stability to result in the same level of osseointegration as compared to unloaded implants and indirectly supports and validates the used osteotomy drill and implant placement protocols. Further newly formed bone was mainly associated to the implant threads indicating that in both healing regimes loads from the implant into bone might mainly be transferred via the screw threads. With regard to the apico-coronal distribution of bone formation coronal BIC values interestingly appeared to be higher when compared with the apical values of loaded implants, which was mainly observed on the buccal aspects of the implants. The corresponding difference for submerged implants was less pronounced. Further from the intergroup comparisons significantly higher buccal (coronal) BATA values were observed for loaded implants compared to the submerged ones. Although this trend of higher BATA and potentially higher BIC was only observed at the buccal aspects, the observed differences might potentially be related to the mechanical stimulation of the crestal bone under loading conditions as also observed in similar study designs performed in the same animal model [24].

Maintenance of the crestal bone level is crucial for peri-implant soft tissue preservation and support [27, 28]. Several factors have been reported to influence the level of peri-implant marginal bone loss. Amongst these factors, the implant position relative to the alveolar crest, the macrodesign of the cervical area (platform-switching vs. platform matching designs), as well as the surface topography at the implant neck might be of specific relevance for the here reported study [29–31]. In this study the implants specifically displayed a platform-switched configuration and were placed subcrestally. Although not statistically significant, transgingivally healed implants displayed consistently lower fBIC values compared to submerged implants. This could be related to differences in mechanical load or possible differences in the exposure of the implants to the microbial environment of the oral cavity [32]. Specifically, the latter difference might be assumed based on the consistent bone overgrowth of subgingivally healed implants in this model. Finally, pCIS values between both groups were comparable and indicated constant vertical bone gain, which was observed for all samples. It is currently unclear if this vertical bone growth was related to the preparation technique of the implantation site (flattening of the alveolar crest). Under consideration of this possible limitation, the study groups did not reveal any significant difference with regard to marginal bone level changes. This would be in line with a recent systematic review by Valles et al., who concluded that subcrestally placed platform switched implants generally display low levels of marginal bone loss and when compared with placement modalities at equicrestal level [29].

Only few studies have been published that have compared the influence of different loading scenarios of dental implants on the osseointegration and marginal bone levels in animal models. The animal model used within this setting has been specifically used previously by Cochran et al. [24] and Stavropoulos et al. [25] who compared the osseointegration between tapered and cylindrical implants after submerged and transgingival healing. It should be kept in mind that the evaluations performed in this study were deliberately designed within a very standardized and previously described model that reflect the clinical workflows only with regard to a very limited set of specific aspects, i.e., drill and placement protocols. Due to these limitations and the large variability of investigational devices and study design as well as due to the novelty and unique characteristics of the here investigated implants, it is further very difficult to perform a meaningful comparison to the results of other preclinical or clinical studies.

In conclusion, the here described comparison of a novel self-cutting tapered implant design after submerged and transgingival, i.e., simulated loading conditions demonstrated equivalent management and maintenance of marginal bone and osseointegration. Osseointegration was not significantly affected by the presence of mechanical load which indirectly validates the osteotomy preparation and implant placement protocols. Within the limitations of the applied model, marginal bone levels were also not affected by the healing and loading regimes. This supports the qualification of the subcrestal placement protocols for immediate loading.
